# 1413. Effect of Automated Identification of Antimicrobial Stewardship Opportunities for Urinary Tract Infections

**DOI:** 10.1093/ofid/ofab466.1605

**Published:** 2021-12-04

**Authors:** Connor Deri, Rebekah Wrenn, Rebekah W Moehring, Justin Spivey, Michael E Yarrington

**Affiliations:** 1 Duke University Hospital, Durham, North Carolina; 2 Duke University, Durham, North Carolina; 3 Duke Center for Antimicrobial Stewardship and Infection Prevention, Durham, NC; 4 Duke University Medical Center, Durham, North Carolina

## Abstract

**Background:**

The treatment of asymptomatic bacteriuria (ASB) does not improve clinical outcomes in most patients and may be associated with an increased risk of adverse events such as *Clostridioides difficile* infection. A best practice alert (BPA) was created to identify patients with possible ASB for antimicrobial stewardship (AS) review. We aimed to determine whether automated identification of ASB improved the timing of stewardship intervention.

**Methods:**

An electronic health record BPA message to inpatient AS pharmacists was activated on 01/19/2021. The BPA identified inpatients with a new antibiotic order with an associated genitourinary indication and a preceding urinalysis with 0 to 5 WBC/hpf. BPAs were reviewed by an AS pharmacist during weekdays and normal business hours. We retrospectively evaluated the impact of the BPA on time from order to stewardship intervention between a cohort of pre-BPA (01/2020 to 12/2020) and post-BPA (01/20/2021 to 04/10/2021) patients. Included patients met the BPA criteria and had an AS intervention within 7 days of the antibiotic order. We specified interventions that were UTI-related. The median time from antibiotic order entry to any AS intervention was compared pre- to post-BPA using the Mann Whitney U test. Rates of UTI-related interventions were compared with Fisher’s Exact test.

**Results:**

327 antibiotic orders met BPA criteria and were analyzed: 245 and 82 in the pre- and post-BPA group, respectively. Groups had similar baseline characteristics (Table 1). A total of 33 (27 UTI-related) pre-BPA group and 24 (17 UTI-related) post-BPA group interventions were documented by the AS team. The median time to any intervention was 28 hours (IQR 18-64.5) in the pre-BPA group compared to 13.5 hours (IQR 3.5-28.75) in the post-BPA group (p = 0.03, Figure). The pre-BPA group had a lower rate of UTI-related interventions compared to the post-BPA group (11.0% vs 20.7%, p = .04).

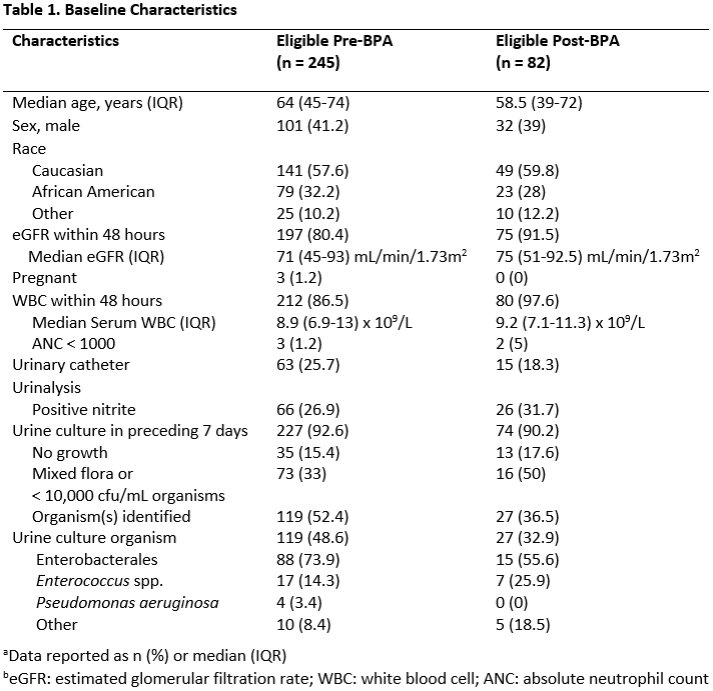

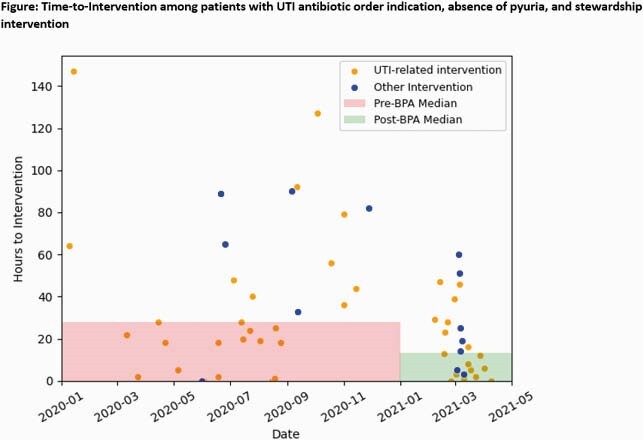

**Conclusion:**

Automated identification of antibiotics targeting UTI with urinalysis showing absence of pyuria reduced the time to stewardship intervention and increased rate of UTI-specific interventions. The use of clinical decision support may aid in efficiency of AS review and syndrome-targeted AS impact.

**Disclosures:**

**Rebekah W. Moehring, MD, MPH**, **UpToDate, Inc.** (Other Financial or Material Support, Author Royalties)

